# Nursing supervision: interfaces with power relations in family health^*^

**DOI:** 10.1590/1980-220X-REEUSP-2022-0034en

**Published:** 2022-08-01

**Authors:** Iramildes Souza Silva, Vivian Aline Mininel, Jaqueline Alcântara Marcelino da Silva

**Affiliations:** 1 Universidade Federal de São Carlos, Programa de Pós-Graduação em Enfermagem, São Carlos, SP, Brazil.

**Keywords:** Nursing, Supervisory, Family Health, Patient Care Team, Power, Psychological, Interprofessional Relations, Supervisión de Enfermería, Salud de la Familia, Grupo de Atención al Paciente, Poder Psicológico, Relaciones Interprofesionales, Supervisão de Enfermagem, Saúde da Família, Equipe de Assistência ao Paciente, Poder Psicológico, Relações Interprofissionais

## Abstract

**Objective:**

To analyze nursing supervision from the perspective of power relations in family health.

**Method:**

An exploratory, descriptive and interpretive research with a qualitative approach. Data were collected through semi-structured interviews with 37 workers from six health teams in a city in the countryside of São Paulo. They were submitted to thematic content analysis, based on the health work process theoretical framework and Foucault’s power category.

**Results:**

Two thematic categories were constructed: *Nursing supervision from the perspective of surveillance and control in relationships of disciplinary power in family health*; *The duality of nursing supervision in family health between oppressive power and positive power.*

**Conclusion:**

The power present in nursing supervision is expressed as control and producer of things, which not only oppresses, but also has positive effects on building healthy work environments, valuing interactions, establishing trust, strengthening teamwork and supporting workers, aspects that result in the promotion of psychological safety in family health.

## RESUMO

**Objetivo:**

Analisar a supervisão exercida pelo enfermeiro na perspectiva das relações de poder na saúde da família.

**Método:**

Pesquisa exploratória, descritiva e interpretativa com abordagem qualitativa. Os dados foram coletados por meio de entrevista semiestruturada com 37 trabalhadores de seis equipes de saúde de um município do interior paulista. Foram submetidos à análise de conteúdo temática, a partir referencial teórico do processo de trabalho em saúde e da categoria poder foucaltiana.

**Resultados:**

Foram construídas duas categorias temáticas: *A supervisão exercida pelo enfermeiro na perspectiva da vigilância e do controle nas relações do poder disciplinar na saúde da família*; *A dualidade da supervisão exercida pelo enfermeiro na saúde da família entre poder opressivo e poder positivo*.

**Conclusão:**

O poder presente na supervisão exercida pelo enfermeiro se expressa como controle e produtor de coisas, que não somente oprime, mas também possui efeitos positivos na construção de ambientes de trabalho saudáveis, com valorização das interações, estabelecimento de confiança, fortalecimento do trabalho em equipe e apoio aos trabalhadores, aspectos que resultam em promoção da segurança psicológica na saúde da família.

## RESUMEN

**Objetivo:**

Analizar la supervisión ejercida por enfermeros en la perspectiva de las relaciones de poder en salud de la familia.

**Método:**

Investigación exploratoria, descriptiva e interpretativa con enfoque cualitativo. Los datos fueron recolectados a través de entrevistas semiestructuradas con 37 trabajadores de seis equipos de salud en una ciudad del interior de São Paulo. Fueron sometidos al análisis de contenido temático, a partir del referencial teórico del proceso de trabajo en salud y la categoría de poder de Foucault.

**Resultados:**

Se construyeron dos categorías temáticas: *La supervisión ejercida por los enfermeros en la perspectiva de vigilancia y control en las relaciones de poder disciplinario en salud de la familia; La dualidad de la supervisión ejercida por las enfermeras en salud de la familia entre el poder opresor y el poder positivo*.

**Conclusión:**

El poder presente en la supervisión ejercida por los enfermeros se expresa como control y productor de cosas, lo que no solo oprime, sino que tiene efectos positivos en la construcción de ambientes de trabajo saludables, valorando las interacciones, estableciendo confianza, fortaleciendo el trabajo en equipo y apoyando a los trabajadores, aspectos que redundan en la promoción de la seguridad psicológica en salud de la familia.

## INTRODUCTION

Nursing supervision in the context of the work process of the family health team (FH) is a suitable tool for changing practices in Primary Health Care (PHC).

The supervision listed in the scope of nurses’ practices has been reaffirmed as a powerful tool, as it impacts work’s care, managerial and educational dimensions and, especially, influences the interactivity of nursing workers with other professional categories^(1)^, including Community Health Workers (CHW)^(2)^.

The study was based on the health work process framework and its constitutive elements – object, instruments, purpose and agents^(3)^. The action of supervising is located in this study as an instrument of nurses’ work, involving interactive social processes, in which different games of interests and disputes coexist, characteristics of the micro-relationships expressed in power relations, between the different categories of workers in FH^(4)^.

There are several developments arising from power relations established among workers in FH, such as the hierarchy of professions and the unequal valuation of the work carried out^(5)^. However, the power relations understood in micropolitics, i.e., in the relational sphere of work, also move a field of forces in the encounters that occur in health work production^(6)^, leading back to the thought that power relations can be productive, based on Foucault’s thinking, whose fulcrum is power and its outcomes.

In this study, it is inferred the need to guide the supervision of nurses inserted in the context of health production and in relationships. For in such a way, Michael Foucault’s works “Discipline and Punish”^(7)^ and “Microphysics of Power”^(8)^ will guide the understanding of nursing supervision from the perspective of power relations in FH.

One of the forms of power exercise investigated in Foucault’s perspective is disciplinary power, constituted by its control devices, characterized in three techniques: hierarchical surveillance, normalizing sanction and examination. In this study, the focus was given to the first, represented in the panopticon’s perception, an architectural project of surveillance similar to a gear, organized under the game of the gaze, which sees, supervises and controls everything, aiming at increasing the possibilities of knowing more about those being watched, to extract from them the best of their trained, docile and economically functioning bodies, but with a decrease in their potential in political terms by establishing obedience^(7)^.

The investigated literature shows that nursing supervision has still been based on the vertical posture of command, with historical roots anchored in hierarchy^(9)^, considered an instrument of asymmetrical power and of inspection^(4)^ and control^(10)^. Therefore, the supervision design exposed refers to the forms described in the disciplinary power and has panoptic traits in its conformation.

However, it must be considered that the characteristics of supervision have undergone changes according to societies’ social and political context and historical moment^(11)^. There is a duality in supervision that points to a management instrument that generates impacts on the team, including those supervised, showing both its operation in strict control and potential to expand health responses in PHC, through reflection and qualification of practices^(12)^.

Nursing supervision reinvention in FH can be established in the context of power relations. Power is capable of producing practices, subjectivities and truths that need to be directed to destroy stereotyped behaviors^(13)^, because the great core of power relations is knowing how to direct them and focus them on a consensual proposal for change^(4)^, in this context, nursing supervision reinvention in FH.

The view of power concentrated in the State as a policy to manage the population’s life and body is shifted to the idea that micropower needs to be considered in relationships between people, in addition to oppressive and destructive power, also as a producer of things^(14)^. Power is not only domination, oppression, it does not only generate obedience, it is productive, it generates resistance, but also positive and, above all, emancipatory^(7)^.

This Foucault’s formulation about power makes it possible to apprehend the object – nursing supervision – from a new perspective. Supervision goes beyond the vigilant function of institutional norms and projects, of strict and coercive controller, focused on economic body production, for a politically reconfigured supervision regarding power relations, starting from the discussion that the notion of domination is insufficient to respond to the conception of productive power, explored in Foucault’s concept of power.

It is based on the assumption that power is always relational, it is exercised in a network, it is not centered on just one pole, and can provide other paths for the exercise of supervision, inserted in nurses’ work, which justifies this investigation. Moreover, there is an important gap in the subject at hand. Publications were found depicting supervision of nurses in general^(1)^, individually for technicians^(12)^, for CHW^(10)^ and studies of power relations in FH^(4,5)^; however, none of them deals specifically with nursing supervision and power relations involving the three professional categories.

This study considers that nursing supervision strongly influences the quality of health care provided by nursing workers and CHW in FH, and the present power relations constitute intervenient in this sense. Thus, it was anchored in the following guiding question: how does nursing supervision in FH occur from the perspective of power relations?

Thus, this study aimed to analyze nursing supervision in FH from the perspective of power relations, focusing on the nursing team and CHW.

## METHOD

### Design of Study

This is an exploratory, descriptive and interpretive research with a qualitative approach, whose focus was nursing supervision in FH from the perspective of power relations. This study adopted the COnsolidated criteria for REporting Qualitative research (COREQ) as a tool to support qualitative approach methods^(15)^.

### Participants

The universe of professional categories involved in this study in FH in the city consisted of approximately 231 members, distributed in 32 teams. The sample of this research consisted of CHW, nurses and nursing technicians, totaling 37 workers. The choice was established by convenience. We included workers of teams who promptly accepted the invitation to participate within the deadline established by the first author of the study, and who met the criterion of working together for at least one year in the same FH team, in the minimum composition modality.

### Local

The study was carried out in a medium-sized municipality located in the geographic center of the state of São Paulo. In this site, the PHC is composed of 24 FH units, in which 32 FH teams work, covering 40% of the population. PHC also has ten Basic Health Units in the traditional model, called the Municipal Health Center (MHC), with 60% coverage, a Health Home Care team (SAD – *Saúde na Atenção Domiciliar*), a PHC supporter and Permanent Health Education Management. After authorization from the PHC coordination, an electronic correspondence was sent to all FH teams. With subsequent signaling of 11 of these, the first author identified those that met the inclusion criteria and a sufficient number of participants to achieve the research objective. The teams that first signaled availability to participate were confirmed, and six were selected because they met the inclusion criteria.

### Data Collection

Data collection took place between April and September 2019, with a script containing guiding questions, which addressed the team’s work process, perceptions about nursing supervision and relationships among FH workers. This script was adapted after the previous application of a pilot questionnaire with five workers, involving the three professional categories studied. The interviews lasted about 50 minutes for nurses and 30 minutes for other team members. They were audio-recorded and fully transcribed, preserving participants’ anonymity.

### Data Analysis and Treatment

The data were fully transcribed by the first author, inserted in Microsoft Word^®^ and submitted to thematic category analysis by Bardin^(16)^, following the steps of text skimming and in-depth reading, vertical analysis of each interview, with the indication of inferences, horizontal analysis by professional group and transversal of all testimonies. Themes were grouped by identified similarities and contradictions, in the light of Mendes-Gonçalves’ health work process frameworks^(3)^ and Michel Foucault’s power category^(7,8)^.

### Ethical Aspects

The research was approved by the university’s Research Ethics Committee, under Opinion 3.229.328/2019, complying with the ethical principles of research involving human beings, according to Resolution 466/12 of the Brazilian National Health Council. Study participants received clarification about the purpose of the research and, upon agreement, signed the Informed Consent Form. The statements were identified through the professional category’s initial letter, such as Community Health Worker (CHW), nurse (N), nursing technician (NT), plus the sequential ordinal number to the interviews.

## RESULTS

Thirty-seven workers from six FH teams participated in the study: 22 CHW, five nurses and ten nursing technicians. Female participants predominated and the mean time working in PHC was seven years. The data analyzed allowed the formulation of two thematic categories: *Nursing supervision from the perspective of surveillance and control in relationships of disciplinary power in family health*; *The duality of nursing supervision in family health between oppressive power and positive power*. In the first category, supervision is explored in the aspect of control in disciplinary power and, in the second, supervision is analyzed under an approach that makes it possible to unveil power as a producer of things, which not only oppresses, but also has its positive effects, such as building healthy work environments, valuing interactions, establishing trust, strengthening teamwork and supporting workers, aspects that result in the promotion of psychological safety in FH.

### Nursing Supervision from the Perspective of Surveillance and Control in Relationships of Disciplinary Power in Family Health

All nurses interviewed showed the perception of disciplinary power centrality in the activity of supervising. The use of control mechanisms was portrayed with emphasis on permanent surveillance of the supervised. Supervision, as a component of this surveillance system, demands the physical presence of nurses to function effectively.

*To know what is happening, it is necessary to be close to the service, otherwise there is no way to know what is being done, just being together to supervise. Presence is important, for example, the coach you spend the whole day with him and the CHW because he is on the street, this is a barrier. That’s why I say that supervision is only possible for those who are here* (N4).

This result also appeared in the testimonies of other workers, when taking supervision as an action that involves watching someone, that the impossibility of monitoring compromises supervision, particularly in relation to CHW.

*The fact that the nursing team works within the unit,* (…) *the CHW, for working on the street, this makes supervision difficult. Inside there is supervision, outside, no. There are CHWs that do not go to the houses, they have to have a more rigorous monitoring; if it really had [supervision], it would solve many cases* (CHW4).

Nurses were uncomfortable in supervising CHW, due to the impossibility of controlling the activities carried out by them, which go beyond the walls of the health unit. This was an important tension point revealed.

*To answer for what CHWs are doing out there, you know? If you’re going home to sleep,* (…) *it anguishes me, not being able to be close* (…)*. So, I think there should be a coordinator* [manager] (…) *apparently that’s what the PNAB* [Brazilian National Primary Care Policy] *is placing. I think it would be fairer in the division of labor. As a nurse, I feel very overwhelmed to deal with what’s going on out there* (N3).

Nurses reinforce another tension in this sense, by disagreeing with the new PNAB guidelines^(1)^, which lists supervising CHWs only in its attributions and not of the other professional categories.

*Supervision in the new PNAB* (…) *is shameful. The term* [attribution] *of supervision was left only to the nurse;* (…) *we have to stay all the time looking if someone* [CHW] *is doing something wrong, doctors and dentists, no* (N4).

Although nursing supervision, identified as strict control, has been mentioned among several professional categories, it was the CHW group that most portrayed this narrative in their work process, through various mechanisms of direct observation, among which were: assessment of the record of the number of visits; assessment of group activities and user signatures, proving these actions; production reports and inspection of workers’ length of stay in the health unit during working hours.

*Supervision was suspicious of whether I was really doing the work, which demotivated me a lot. The way* [supervision] *expressed itself, how the numbers of visits were asked of me in the assessment* (…) *“If you don’t do it* [the number of standardized visits], *supervision will be able to punish you”* (CHW9).

*Supervising means having to keep an eye on everything and see if everything is in order* [example], *if I am not visiting, knowing why I am not* (CHW6).

Another data presented in the reports indicated that one of the control devices for the exercise of supervision involves assisting in a room strategically located in the physical space of the unit as a measure to ensure that there is no behavior by the supervised that is incongruent with the standards agreed upon for serving the population.

*In the supervision of a NT* [nursing technician], (…) *I stay in a place that I think is strategic. I work in the room in front of a room where the girls* [nursing technicians] *are at the reception and* (…) *because they do a lot of care and I end up knowing how they care and, later, I end up making some change and intervention* (N2).

The use of an indirect control mechanism in supervision of both CHW and nursing technician was also pointed out.

*In the case of NT, like it or not, they are under “our eyes”, even though they are in another room, but in the same physical space. The supervision of CHW is a little more difficult, because he is on the street, and we spend a lot of time in the unit* (N1).

The centralization of power *in* and *by* nurses for supervision was described as unfavorable, by decreasing teamwork power.

*Everyone puts her* [supervisor] *at the center of everything* (…) *it’s a weakness in teamwork, everyone runs just to her, and when she’s not,* (…) *you have to wait until the next day for her to tell the employee to do what should have already been resolved* (…) *she has to have a dialogue, she centralizes power more in her* (NT4).

Supervised participants mentioned that supervision, instead of being a tool of power at the service of strict control and domination, should have more flexibility to bring supervisor and supervisee together in effective actions in FH. It was recognized that the rigidity in this relationship drives power imbalance and may negatively impact the final result of work.

*Oversight should not be an instrument of power; the supervisor would be able to “convey” proximity for us to work properly. If you supervise in a brusque, rigid way, people can’t work properly; under tension, it is not nice to work. I’ve felt suffocated and watched at all times. This power relationship can affect everyone, disrupting and unbalancing; the work cannot be done in the best way* (CHW2).

### The Duality of Nursing Supervision in Family Health Between Oppressive Power and Positive Power

Nursing supervision was evidenced as a tool that raises both oppression, expressed as fear, insecurity and disagreements in work relationships, as well as the instrument for positive advances, which were revealed in supervision as a power capable of assertively influencing the work process in FH, either by the dialogic relationship it establishes or by favoring a healthy environment, according to experiences described.

*I know a lot of people who cry, hurt and depressed because of the destructive power of supervision* [vertical] (…)*. But the experience I have now in this team is of a positive power, to lead, to talk, to go together, to train, to direct and to help* (NT2).

*In my trajectory in that time of oppression of supervision, the team was disunited; it was afraid, it could not perform the service safely; a trapped team. At the moment, working while being heard is different, the service takes off, you get feedback and you are mentally healthy* (NT6).

*I had a supervisor who worked with “an iron fist”; it was a lot of demand, it generated irritation and discontent. Here, I don’t feel that, for me it’s a help and incentive to improve my work, “Come on, we have to make groups”* [the nurse speaks to the CHW] (CHW7).

In this sense, the statements also highlighted that supervision promotes psychological safety in the workplace, a factor that influences the mitigation of illness at work with the promotion of workers’ well-being.

*Supervisors are also psychologists; they listen to employees and help us to stop, set limits, because I absorb a lot of problems from the work process and they show that we can’t solve everything, otherwise it creates a lack of control in my life and, sometimes, without even saying anything, they already help. It is very important to be supervised. I idealize and experience it* (NT9).

*Supervisors are people who provide security at work and support in difficult situations* (CHW8).

Still in this direction, the contribution to ensuring safe care was provided by supervision, by carrying out interlocution and socialization of intra-team care protocols, as well as by fostering a relationship of trust based on supervision’s ethical attitude.

*If I didn’t have supervision in primary care, it would be like a car without an engine, it doesn’t work; there has to be a nurse to lead so that the team feels safe, to help us understand certain details of the protocols that are passed to her* [supervisor]; *this makes the team more integrated, with more autonomy to act* (CHW8).

Carrying out the work without the fear that the assessment will result in punishment is defended by a participant who experiences a more flexible supervision, which has as a consequence the freedom to act, unlike the report of other participants about performance assessment based on punishment.

*Supervision is a performance assessment with the objective of improving, of achieving some established goal. Here, supervision happens in a positive, non-punitive way, the model that is done here leaves us freer* (CHW1).

Despite the positive results presented in this category, nurses demonstrated to recognize that supervision in the problematizing educational approach still needs to advance.

*There is no time* (…)*. I would like to sit down, listen, see what the possibilities are and ask* [the supervised]: *“what is your affliction, because you can’t, for example, perform a mental health reception with a patient, do you have a problem, let’s talk about it”?* (N1).

## DISCUSSION

In this study, nursing supervision, inserted in the partial composition of work and as a relational practice, proved to be a predominance of the logic of strict control, characterized by the need for permanent surveillance over those supervised. Supervision effectiveness, therefore, seems to be conditioned to nurses’ physical gaze, which functions as a power device. Device is understood as the correlation between institutional, administrative and physical power techniques, as schematized in the panopticon apparatus described.

The panoptic principle^(7)^ induces workers to adapt to what is standardized for their function, in order to be accepted exemplarily in the health team, especially by supervision, or, ultimately, they are free from suffering punitive actions, especially regarding assessment processes. Punitive supervision has been discouraged, considering its deleterious effects on health work outcomes^(17)^.

In the perception of nurses, when comparing nursing technicians’ supervision with CHW’s supervision, in a way, it is easier to supervise the former, because they are under the same roof, unlike CHWs, which act most of the time in extramural activities. According to the interviewees’ opinion, the supervision of this worker seems to have less space in this nurses’ attribution, not only because of the geographic barrier itself, but because nurses do not have control over CHWs’ work, because they cannot supervise them.

It is important to recognize the role played by CHWs in the enrichment of clinical actions in the care provided in FH, as it gathers and shares fundamental details for a broader understanding of users’ health needs. However, hegemonic disputes between the various professions tend to delegitimize workers’ knowledge, compromising accountability in PHC^(6)^.

According to the results revealed, control devices in supervision appeared directly and indirectly, the former more frequently in relation to CHWs’ work, translated into demands for productivist goals, such as complying with the minimum number of daily home visits, carrying out group activities and their respective supporting documents and indirectly, when nurses check the visits with users. Indirect control devices were more strongly identified in relation to nursing technicians. The panoptic evidence observed in supervision suggests that one of the ways to inspect would be to stay at a fixed point in the health unit, strategically chosen, so that the supervisory look and hearing could assess what happens in nursing technicians’ work process regarding the way they assist users; however, this does not ensure that nurses know the totality of nursing technicians’ actions. It is emphasized that these have a relevant role in PHC, particularly in user reception^(12)^.

CHW’s work operates in the productivity logic, and in their supervision, bureaucratic control prevails, with a focus on the regulation of actions, assessed by mechanisms such as record assessment and meetings for accountability^(10)^.

In this context, nurses question the fact that the last update of the PNAB^(2)^ states that nurses must supervise CHWs, disregarding other categories, such as doctors and dentists This is an aspect that tensions and moves team power relations, generating nurses’ dissatisfaction due to the lack of co- responsibility of the other categories mentioned in relation to CHW’s work.

In order to control human work, several methods are used so that it develops characteristics similar to those of a machine^(18)^. Localized supervision in hegemonic management rationality in health adopts methods of direct and indirect control, in an attempt to shape workers and regulate work. This rationality is supported by political, economic and scientific arguments aimed at reducing workers to a functional tool through protocols that determine convenient conducts and behaviors^(18)^.

Authors argue that, in FH, bureaucratic demands are voluminous and bureaucracy transforms norms and rules into sacred. It is not questioned whether they exist, but the priority they occupy in the organization of work and the rigidity with which they are charged to workers, who are sometimes trained to act and meet institutional interests^(19)^.

Another data analyzed concerns power centralization in and by nurses for supervision. This condition may be a consequence of the team’s omission, demonstrated in the little proactive attitude in everyday decision-making processes. It seems that the team does not feel like having co-responsibility for what happens in the work process, maintaining the status quo; otherwise, the team would have to position itself in several aspects, including the exposition of what they think, which would place its elements in the arena of power relations, with multiple unfolding, such as conflict establishment^(20)^.

It is assumed that nurses concentrate power as a reproduction of the very structure in which supervision is traditionally inscribed, in strict control, with an emphasis on verticalization^(21)^, as discussed above, which emphasizes work from the perspective of productivity.

The study also evidenced nurses’ recognition of the gap present in supervision, in the sense of education for problematizing practice in FH. There are numerous existing crossings, which disfavor the educational dimension as a priority in health. One of the allegations was nurses’ lack of time to implement the analysis and reflection of the cases in the work process, so that the supervised is able to act in a contextualized way in user service. A study that questions the reason for nurses’ difficulty in adding an educational perspective to supervision corroborates this discussion. Work overload is identified as one of the factors that limit the activity of supervising, together with the need for investments in the training of professionals, appreciation of comprehensiveness as a model of care organization and participatory management models, which favor the collective construction and permanent education actions in health at work^(1)^.

The conditions pointed out result in damage to the work in integrated teams. Centralizing power means increasing or maintaining inequalities among workers, it implies emptying the problem-solving capacity, which is provided by knowledge and responsibility sharing, and power democratization in the team’s experience. The idea of power based on someone is rejected, of seeing workers as passive depositaries, mere victims of the exercise of power or its structures^(22)^. We work with the power microphysics conception, moving in the relationships among subjects, in their circularity and capillarity throughout the social body, producing things^(8)^.

From this point of view, although strict control in nursing supervision was prevalent in the findings, the data also converged to other characteristics present in supervision, which place this activity as an instrument of positive advances in FH’s work towards the team’s psychological safety and the recognition of the need to move in the educational direction.

Taking the concept of power from a perspective that transcends oppression, supervision was recognized in this study as an instrument of power that makes other moves in the team’s work process, which perhaps do not have enough power to disentangle supervision from this configuration of control, but point to an articulating practice of dialogue, considered one of the most fertile ways to reduce power imbalance among workers, with repercussions on the production of better health results as work purpose. Overcoming fragmented and dominant models of health care is made possible by dialogue and reflection among team members^(23)^. Nursing supervision is relevant in this sense; however, one of the shortcomings in nurses’ training process, which compromises the exercise of supervision, is this gap in the dialogic perspective^(1)^.

Another finding in the direction of supervision as a positive power is related to its contribution to the vocalization of the different actors in the team, in their discomforts, anguish and work difficulties, notably promoting workers’ mental health individually and, collectively, the work environment’s psychological safety, seen as shared belief among the health team that they are safe to take on the uncertainties and challenges of work^(24)^. Conversely, absence of psychological safety reinforces the asymmetries of power in the team, as it inhibits its components from speaking, silencing them^(24)^.

In this study, the psychological safety fostered by nursing supervision also approached the guarantee of safe care in FH. Nurses as supervisors were evidenced as those who make institutional projects happen to improve care for the population, when they make efforts for intra-team socialization and in articulation with central management in this sense. A study in which the PHC team is committed to service quality, based on this nurses’ action, contributed to this result^(25)^.

As a limitation of this study, the impossibility of generalizations considering the number of teams analyzed stands out. It is understood that supervision, from the perspective of power relations in FH, should be explored in different PHC contexts, based on other theoretical and methodological frameworks, in order to deepen the investigated theme’s complexity.

Due to the importance that nursing supervision has in FH teams’ work, this study made it possible to highlight aspects that interfere in this attribution, sometimes making it reproduce the logic of strict control, but also enabling other positive productions for health outcomes, health promotion in the workplace and nursing knowledge.

## CONCLUSION

This study shows that nursing supervision in FH still preserves characteristics of strict control, with traces of panoptic surveillance, both in the way nurses conceive supervision and in the mechanisms used by them in this attribution.

It is discussed the need to displace nursing supervision from this place in which it was historically inscribed, in the logic of control, for a practice that provides other ways of relating to the supervised and other team members. Several factors are involved in this change, among them, the need for co-responsibility in the decisions of what happens in the team and the democratization of power in the daily work, built mainly from dialogue.

Thus, the possibility of repositioning supervision in this intersubjective dynamics and analyzing it as a dialogical practice to produce advances in health work stands out. When supervising, nurses collaborate to building healthy work environments, valuing interactions, establishing trust, strengthening teamwork and supporting workers, aspects that result in the promotion of psychological safety in FH.

In this sense, the findings of this study also show that the power relations analyzed in the supervision concept performed by nurses in FH can influence the set of factors present in the team’s work, modify the concept of supervision and, consequently, transform it.
